# The use of frozen embryos and frozen sperm have complementary IVF outcomes: a retrospective analysis in couples experiencing IVF/Donor and IVF/Husband

**DOI:** 10.1186/s12884-022-05088-x

**Published:** 2022-10-18

**Authors:** Yong Zhu, Feng Zhang, Hua Chen, Xiaoxi Sun, Feng Jiang

**Affiliations:** 1 Obstetrics and Gynecology Hospital, Institute of Reproduction and Development, Fudan University, Shanghai, China; 2 Shanghai Ji Ai Genetics and IVF Institute, Obstetrics and Gynecology Hospital, Fudan University, 200011 Shanghai, China

**Keywords:** Cryopreservation, IVF outcome, Sperm, Embryo, Accumulative effect

## Abstract

**Background:**

The cryopreservation of sperm or embryos has been an important strategy in the treatment of infertility. Recently studies have revealed the outcomes after IVF (in vitro fertilization) treatment for single-factor exposure either to frozen sperm or embryos.

**Methods:**

This retrospective study was to uncover the exposure to both frozen sperm and embryo effects using IVF/H (in vitro fertilization using husbands’ fresh sperm) or IVF/D (in vitro fertilization using donors’ frozen sperm) treatment.

**Results:**

The results showed the clinical pregnancy rate (CPR), live birth rate (LBR) and low birth weight rate (LBW) increased to 63.2% (or 68.1%), 61.1% (or 66.4%) and 15.8% (or 16.2%) after using frozen embryo transfer within Group IVF/H (or Group IVF/D). After using frozen sperm, the high-quality embryo rate (HER) increased to 52% and baby with birth defect rate (BDR) reduced to 0% in subgroup D/ET comparing to subgroup H/ET. While the fertilization rate (FER), cleavage rate (CLR), HER and multiple pregnancy rate (MUR) reduced to 75%, 71%, 45% and 9.2% in subgroup D/FET comparing to subgroup H/FET. Finally, our study found accumulative frozen gamete effects, including both sperm and embryos, led to the significantly increasing in the HER (*p* < 0.05), CPR (*p* < 0.001), LBR (*p* < 0.001) and LBW (*p* < 0.05) in subgroup D/FET comparing to subgroup H/ET.

**Conclusion:**

The use of frozen embryos and frozen sperm have complementary IVF outcomes. Our findings highlighted the parent’s distinguished frozen effect not only for clinical studies but also for basic research on the mechanism of cellular response adaptations to cryopreservation.

## Introduction

To keep the division of embryo the same pace with the growth of the endometrium, the transferring of frozen embryos have been widely used in assisted reproductive technology (ART). Since the first successful report of frozen embryo transfer (FET) [1], the cryopreservation of embryos has been an important strategy in the treatment of infertility. FET strategies contribute an additional 25–50% chance of pregnancy for couples who have cryopreserved embryos [2–4]. However, FET is not free from the risk of a higher multipregnancy rate (MPR) and low birth weight rate (LBW), even though the live birth rate (LBR) of frozen–thawed embryos is usually higher than that of fresh transferred embryos [5–8]. On the other hand, male due to azoospermia or sperm retrieval difficulties on the day of egg retrieval in vitro fertilization with frozen spermatozoa is used to treat female who might also have tubal lesions or those experiencing failure of prior artificial insemination with donor semen (AID) cycles [9, 10]. LBW Meanwhile, there is a matter of debate on the clinical outcomes caused by alterations in the DNA integrity of semen following cryopreservation [11, 12]. Indeed, the baby with no birth defect rate following pregnancies after IVF/D was not different (97.3% vs. 97.4%) [13] after IVF with fresh husband spermatozoa, but the clinical pregnancy rate (CPR) per transfer was higher after using frozen donor semen than that after using the husbands’ semen (27.6% vs. 21.9%, respectively)[13] Above all, taking advantage of freezing sperm, eggs or embryos is just like a double-edged sword for IVF outcomes, so doctors who use it need to be cautious.

Nowadays, the practical ART will also meet the clinical treatment of one couple not only need to freeze sperm, but also suitable for freezing embryos. There still have been less studies of this double-factors freezing on the IVF outcomes including fertilization, pregnancy, and child birth. Most of published studies are incomprehensive, they are retrospective or refer to cases involving either frozen embryo transfer or frozen donor spermatozoa. For this article, we cover the findings of both freezing embryos transfer (FET) and IVF/D. As far as ART procedures are concerned, FET and IVF/D lead to some specific questions requiring answers: (i) the effects on the pregnancy and neonate characteristics of single-factor exposure to frozen embryo transfer; (ii) the effects on the fertilization, pregnancy and neonate characteristics of single-factor exposure to frozen donor semen; and (iii) the influence of the duration of sperm and embryo cryopreservation on pregnancy and newborn health. Methods.

## Methods

This study was approved by the Ethical Committee of the institutional review board of the Ethical Committee of the Obstetrics and Gynaecology Hospital of Fudan University and written informed consent was obtained from all couples. All methods were performed in accordance with the relevant guidelines and regulations.

We retrospectively analyzed the IVF treatment of couples experiencing infertility with frozen-thawed donor sperm (Group IVF/D) or fresh husband sperm (Group IVF/H) at Shanghai Ji Ai Genetics and IVF Institute (Jan. 2013-Feb. 2019), after which we followed up until the birth of the baby. For Group IVF/D, the donor’s inclusion criteria were normal sperm parameters above World Health Organization guidelines [14]. For Group IVF/H, the husband’s inclusion criteria were also normal sperm parameters according to World Health Organization guidelines [14]. The inclusion criteria for women in both groups were those undergoing their first IVF-ET cycle caused only by tubal obstruction factors. Women with premature ovarian failure, polycystic ovarian syndrome, chromosome abnormalities, habitual abortion and other diseases that cause infertility were all excluded from this study. In addition, male patients with anti-sperm antibodies, high DNA fragmentation index, non-ejaculation, retrograde ejaculation, chromosome abnormalities and other diseases that cause infertility were also excluded.

The processing of the semen sample treatments was as follows: According to the World Health Organization guidelines [14], donors’ semen samples were obtained by masturbation. After liquefaction of the fresh ejaculate, the specimens’ characteristics were evaluated, such as volume, count and motility. The qualified donors followed the National Sperm Bank reference for semen parameters (before freezing: volume ≥ 2.0 ml, concentration ≥ 60 million/ml, progressive motility ≥ 60%, normal sperm morphology ≥ 70%; after thawing: concentration ≥ 15 million/ml, progressive motility ≥ 32%, normal sperm morphology ≥ 4%). On the day of egg collection by uterine surgery, the semen samples were thawed in a water bath at 37 ℃. Then, the semen sample was treated by the density gradient method (45% and 90% gradient solution, Vitrolife, Gothenburg, Sweden), by which the samples were centrifuged at 500 g for 20 min. After removing the supernatant, the precipitate was washed with washing solution (Vitrolife, Gothenburg, Sweden) and centrifuged at 300 g for 10 min. For the husband, all the semen samples were obtained by masturbation on the day of egg collection by uterine surgery, and the fresh sperm were used in the IVF-ET cycles.

### The processing of ovulation, fertilization and embryo transfer were as follows

controlled ovarian hyperstimulation (COH) used a gonadotropin-releasing hormone agonist protocol (the following was a shortened version of the long protocol) or a GnRH antagonist protocol, both of which are effective in blocking a premature LH surge [15, 16]. Generally, for the long protocol, a GnRH agonist (Triptorelin Acetate, Ipsen Pharma Biotech, France) was administered by subcutaneous injection daily starting from the luteal phase of the menstrual cycle for 10–14 days, and then ovarian stimulation with rFSH (Gn-F, Merck Serono SA Aubonne Branch, Switzerland) commenced. For the GnRH antagonist protocol, ovarian stimulation began on the second day of the menstrual cycle, and on the fifth day, antagonist (Cetrorelix Acetate, Cetrotide, Serono Labs Inc., Switzerland) administration started. Once the leading two follicles reached 18 mm or larger in diameter, the hCG administration was ejected as a trigger on the same day. Thirty-five to forty hours later, a doctor punctured the follicles and collected the eggs with the guidance of an ultrasound instrument. The sperm and oocytes (10000:1) were added to 0.1 ml of prebalanced embryo culture medium into four-well plates covered with mineral oil. The evaluation of embryo quality was performed on the 3rd day after fertilization. The embryos were divided into four levels according to the characteristics of blastomeres, such as the number, the morphology and fragments. Grade I and Grade II embryos were defined as high-quality embryos, and the other grades were defined as low-quality embryos [17, 18]. When the number of high-quality embryos was two or lower and the woman was younger than 35 years old, the embryos were frozen for transfer. All transferred embryos were 4-cell embryos. The frozen embryos were thawed according to the rapid recovery method of vitrification. Embryos with a recovery rate above 50% could be used for transfer. After embryo transfer, the patients were followed up until birth.

### Grouping and statistical analysis

**Fig. 1:**
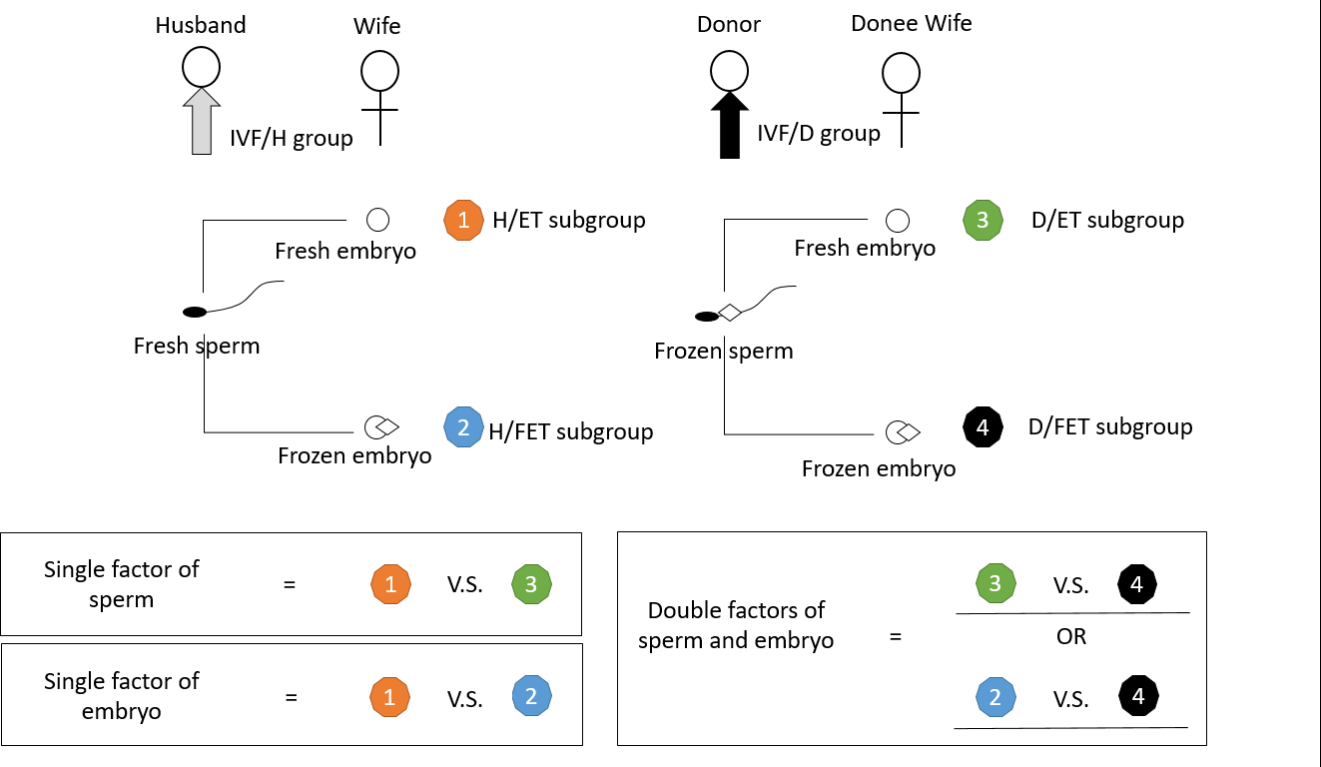
The research grouping flow chat

According to the treatment of embryo transfer, Group IVF/D was divided into the D/ET subgroup (treated with frozen donor sperm and Fresh embryo transfer) and the D/FET subgroup (treated with frozen donor sperm and frozen embryo transfer). Group IVF/H was also divided into the H/ET subgroup (treated with Fresh husband sperm and Fresh embryo transfer) and the H/FET subgroup (treated with Fresh husband sperm and frozen embryo transfer). The detail of the research grouping flow chat is shown in Fig. 1.

The female-related factors, including age, GnRH injection days and dosage, the estrogen secretion peak of egg collection, endometrium thickness, the number of eggs retrieved, the number of fertilizations, the number of effective embryos, the number of high-quality embryos, etc., were followed up and analyzed by SPSS 19.0 software (SPSS Inc., Cambridge, MA, USA). First, all the parameters were checked by a normal distribution test (Kolmogorov–Smirnov model and Shapiro–Wilk model). Then, the medians (first quartile, third quartile) of the continuous parameters were calculated. Finally, the variation in clinical outcomes within or between the IVF/D or IVF/H groups was compared using the nonparametric test (Kruskal–Wallis model) or chi square test for independent samples (*p* < 0.05).

## Results

### Participants and grouping

A total of 860 couples undergoing IVF-ET treatment were included in the analysis, including 573 couples with husband sperm (Group IVF/H) and 287 couples with frozen donor sperm (Group IVF/D). According to whether or not frozen embryo transfer was performed, the IVF/H group was divided into the H/ET subgroup (133 couples treated by IVF/H and fresh embryo transfer) and the H/FET subgroup (440 couples treated by IVF/H and frozen embryo transfer); moreover, the IVF/D group was also divided into the D/ET subgroup (49 couples treated by IVF/D and fresh embryo transfer) and the D/FET subgroup (238 couples treated by IVF/D and frozen embryo transfer) (detailed in Fig. 1).

### The baseline characteristics of the couples

Except for the female BMI in the FN subgroup, the variables were nonnormally distributed among the other subgroups. Thus, using the nonparametric test (Kruskal–Wallis model), we found that the median endometrial thickness (10 cm vs. 9 cm, *p* = 0.015), oocytes retrieved (8 vs. 11, *p* = 0.000) and MII oocytes (6 vs. 10, *p* = 0.000) were differentiated within Group IVF/H, but we found that only female age (30 years vs. 28 years, *p* = 0.002) was differentiated within Group IVF/D. There were no significant differences (*p* > 0.05) within Group IVF/H or Group IVF/D, including the male age, semen parameters and female BMI (detailed in Table 1).

**Table 1:** The basic characteristics of the 860 couples in one IVF/H (or IVF/D) cycle

Baseline Parameters	Group IVF/H		***P value*** (H/ET vs. H/FET)	Group IVF/D		***P value*** (D/ET vs. D/FET)
	H/ET^1^ subgroup	H/FET^2^ subgroup		D/ET^3^ subgroup	D/FET^4^ subgroup	
	Median [first quartile, third quartile]			Median [first quartile, third quartile]		
Inclusion Samples (n)	133	440	/	49	238	/
Male Age (husband or donor) ^a^	32 [29, 34]	32 [29, 34]	0.731	23 [22, 26]	23 [21, 27]	0.967
Semen Volume (Ml) ^a^	3 [2, 3]	3 [3]	0.348	2 [2, 2]	2 [2, 2]	0.608
Semen Concentration (10^6/ML) ^a^	56 [48, 64]	54 [48, 62]	0.734	46 [43, 53]	47 [43, 51]	0.974
Semen (PR + NP) (%)^a^	56 [50, 60]	54 [50, 60]	0.931	46 [43, 54]	48 [43, 52]	0.574
Female Age ^a^	33 [30, 35]	32 [30, 35]	0.644	30 [28, 35]	28 [26, 32]	0.002**
Female BMI (kg/m^2^) ^a^	21 [24, 25]	21 [26, 27]	0.862	22 [20, 23]	21 [20, 24]	0.862
Endometrial Thickness (cm) ^a^	10 [8, 12]	9 [7, 11]	0.015*	10 [8, 12]	10 [8, 12]	0.805
Oocytes Retrieved (n) ^a^	8 [4, 11]	11[8, 16]	0.000***	11 [8, 15]	11 [8, 15]	0.590
MII Oocytes (n) ^a^	6 [4, 10]	10 [6, 14]	0.000***	9 [7, 13]	9 [6, 12]	0.721
MII Oocytes (%)^a^	93 [78, 100]	91 [82, 100]	0.660	81 [68, 96]	86 [73, 96]	0.503

Note:

^1^: H/ET subgroup = using fresh sperm and fresh embryo transfer;

^2^: H/FET subgroup = using fresh sperm and frozen embryo transfer;

^3^: D/ET subgroup = using frozen sperm and fresh embryo transfer;

^4^: D/FET subgroup = using frozen sperm and frozen embryo transfer;

^a^: Nonparametric test (Kruskal–Wallis Model)

* *p* < 0.05 **p < 0.01 ***p < 0.001

### Maternal factor exposure to frozen embryo transfer: the comparison of clinical outcomes within group IVF/H or group IVF/D

Using the nonparametric test (Kruskal–Wallis Model), the outcomes of fertilization and embryo transfer were compared within Group IVF/H and Group IVF/D (see Table 2). Before embryo transfer, the median of all the fertilization outcomes in the H/FET subgroup was higher than that in the H/ET subgroup. The median [first quartile, third quartile] fertilization rate (%), cleavage rate (%) and high-quality embryo rate (%) were 80 [67, 91], 100 [80, 100] and 57 [36, 78], respectively, in the H/FET subgroup. In contrast, there was no significant difference between the D/ET and D/FET subgroups. After frozen embryo transfer, the CPR and LBR were significantly higher in the H/FET subgroup than in the fresh embryo treatment subgroup and the H/ET subgroup (63.2 vs. 39.8, *p* = 0.000; 61.1 vs. 39.1, *p* = 0.000; 14.8 vs. 6.7, *p* = 0.016, respectively). However, the LBW was significantly higher in the H/FET subgroup than in the fresh embryo treatment subgroup in the H/ET subgroup (16.2 vs. 4.9, *p* = 0.021). In another IVF/D group, the CPR and LBR were significantly higher in the D/FET subgroup than in the fresh embryo treatment subgroup in the D/ET subgroup (68.1 vs. 49.0, *p* = 0.017; 66.4 vs. 47.0, *p* = 0.010, respectively). Otherwise, the BDR was significantly higher in the D/FET subgroup than in the fresh embryo treatment group in subgroup D/ET (1.0 vs. 0.0, *p* = 0.000).

**Table 2:** The IVF outcomes of the 860 couples in one IVF/H (or IVF/D) cycle

Outcome Indicators	Group IVF/H		***(Χ*** ^***2***^ ***)*** ***P value*** (H/ET vs. H/FET)	Group IVF/D		***(Χ*** ^***2***^ ***)*** ***P value*** (D/ET vs. D/FET)
	H/ET^1^ subgroup	H/FET^2^ subgroup		D/ET^3^ subgroup	D/FET^4^ subgroup	
	Median [first quartile, third quartile]			Median [first quartile, third quartile]		
After Fertilization						
Fertilization Number (n)^a^	5 [3, 8]	8 [5, 11]	0.000***	8 [6, 12]	8 [5, 11]	0.342
Fertilization Rate (%)^a^	71 [50,89]	80 [67,91]	0.001**	67 [57,88]	75 [57,84]	0.822
Cleavage Number (n) ^a^	4 [2, 8]	8 [5, 11]	0.000***	8 [6, 11]	7 [4, 11]	0.309
Cleavage Rate (%)^a^	67 [50,88]	100 [80,100]	0.000***	67 [57,88]	71 [55,82]	0.667
High-Quality Embryos (n) ^a^	2 [1, 5]	4 [3, 7]	0.000***	6 [3, 8]	4 [2, 7]	0.105
High-Quality Embryos Rate (%)^a^	33 [10,54]	57 [36,78]	0.000***	52 [33,67]	45 [28,60]	0.070
After Transfer						
Biochemical Pregnancy Rate (%)^b^	0.0	1.4	(2.377) 0.123	2.0	0.4	(1.542) 0.214
Clinical Pregnancy Rate (%)^b^	39.8	63.2	(22.789) 0.000***	49.0	68.1	(5.734) 0.017*
Live Birth Rate (%)^b^	39.1	61.1	(20.134) 0.000***	47.0	66.4	(6.598) 0.010*
Multipregnancy Rate (%)^b^	6.7	14.8	(5.820) 0.016*	14.3	9.2	(1.137) 0.286
Miscarriage Rate (%)^b^	0.8	2.0	(0.997) 0.318	2.0	2.1	(0.001) 0.979
Total Baby Sex Ratio (%)^b^	69.4	114.1	(3.129) 0.077	121.4	82.0	(1.036) 0.309
Low Birth Weight Rate (%)^b^	4.9	16.2	(5.286) 0.021*	19.4	15.8	(0.029) 0.806
Baby with Birth Defect Rate (%)^b^	1.6	1.5	(0.007) 0.933	0.0	1.0	(216.712) 0.000***

Note:

^1^: H/ET subgroup = using fresh sperm and fresh embryo transfer;

^2^: H/FET subgroup = using fresh sperm and frozen embryo transfer;

^3^: D/ET subgroup = using frozen sperm and fresh embryo transfer;

^4^: D/FET subgroup = using frozen sperm and frozen embryo transfer;

^a^: Nonparametric Test (Kruskal–Wallis Model)

^b^: chi square test for independent samples

* *p* < 0.05 **p < 0.01 ***p < 0.001

### Paternal factor exposure to frozen sperm fertilization: the comparison of clinical outcomes between Group IVF/H and Group IVF/D

**Fig. 2:**
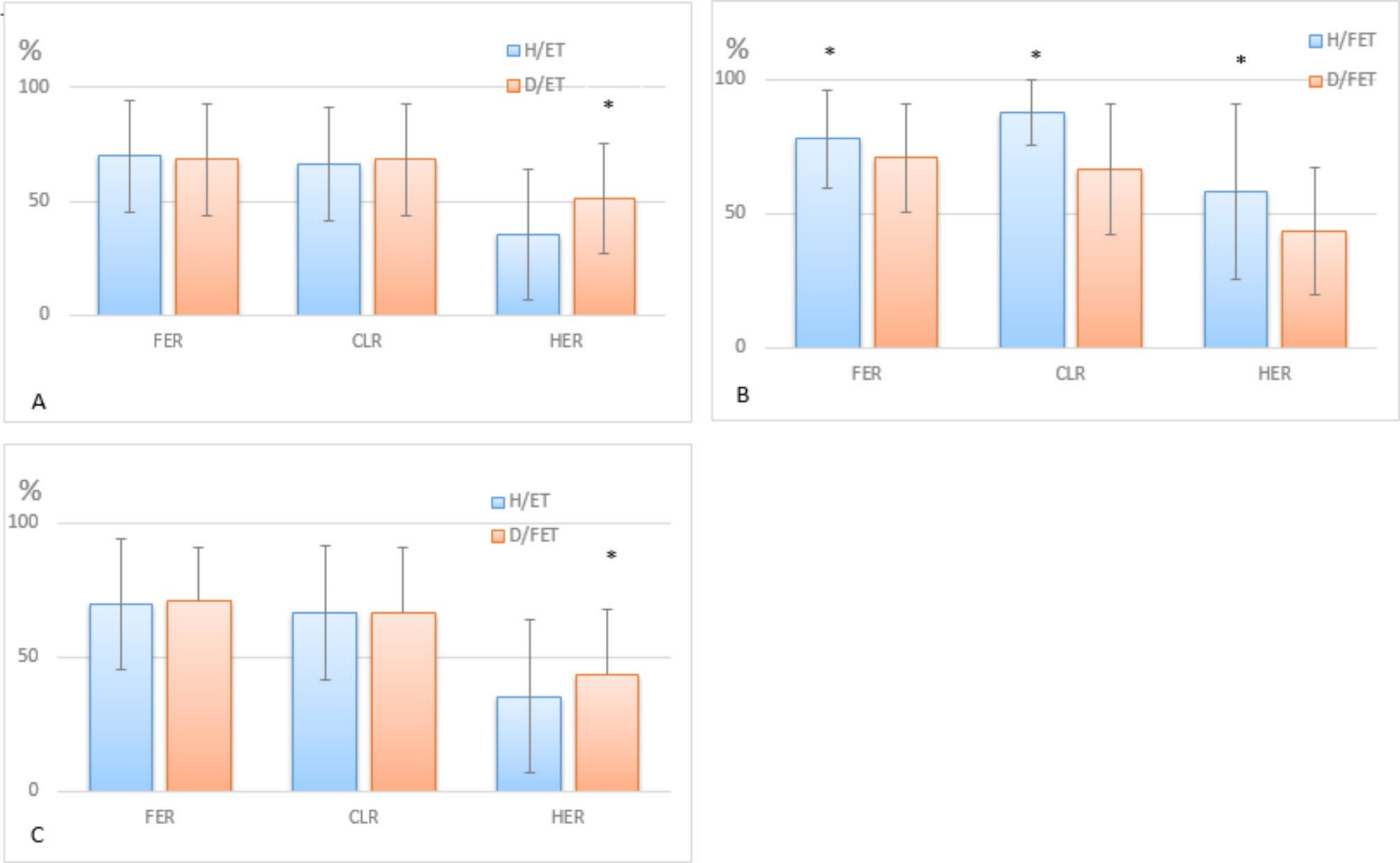
The comparison of fertilization outcomes between Group IVF/H and Group IVF/D. The values of the FER, CLR and HER in subgroups H/ET and H/FET are shown in the separated blue histogram and those of the D/ET and D/FET subgroups are shown in the red histogram. Note: FER = fertilization rate; CLR = cleavage rate; HER = high-quality embryo rate; * *p* < 0.05 **p < 0.01 ***p < 0.001

**Fig. 3:**
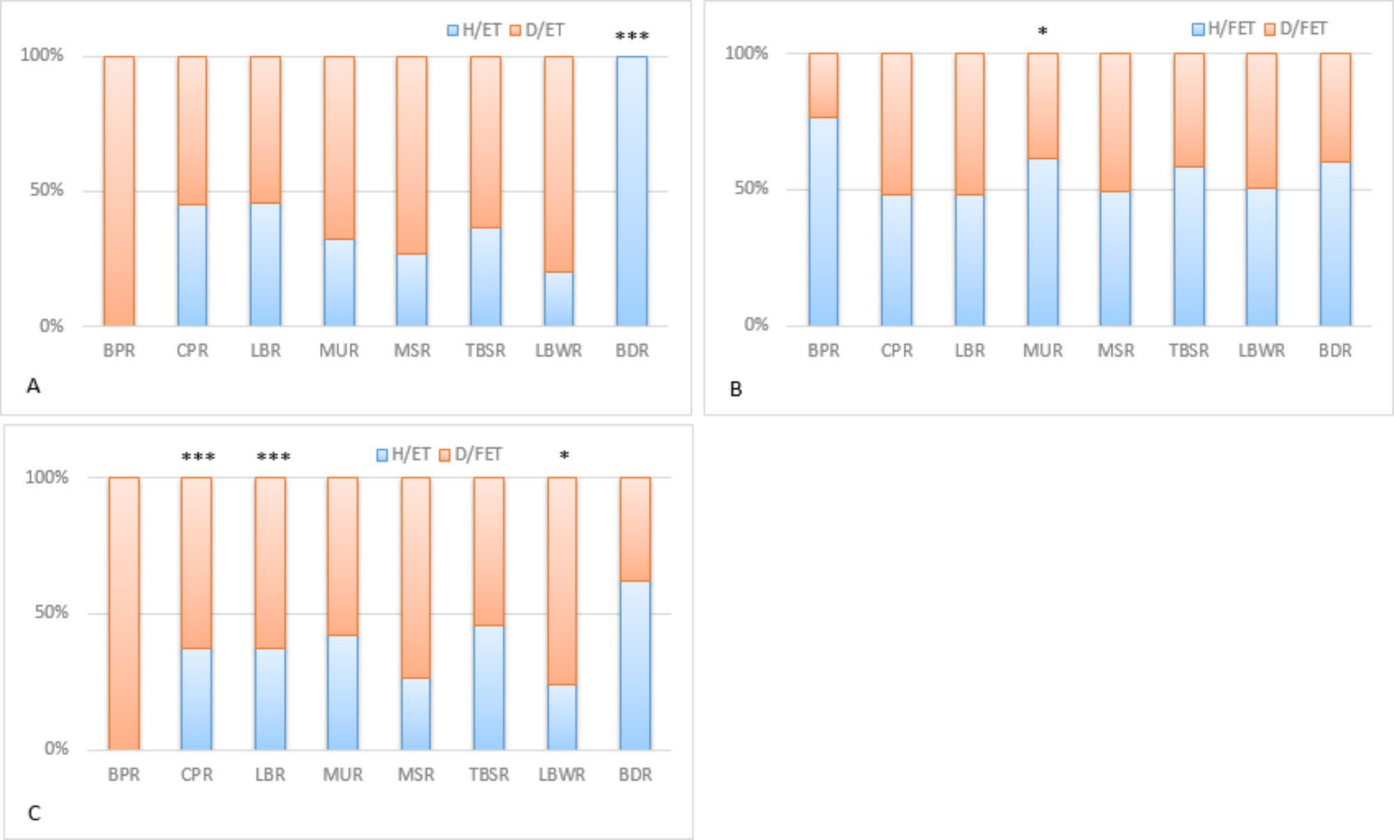
The comparison of embryo transfer outcomes between Group IVF/H and Group IVF/D. The proportions of the BPR, CPR, LBR, MUR, MSR, TBSR, LBW and BDR in the H/ET and H/FET subgroups are shown in the combined blue histogram and those of the D/ET or D/FET subgroups are shown in the combined red histogram. Note: BPR = biochemical pregnancy rate; CPR = clinical pregnancy rate; LBR = live birth rate; MUR = multipregnancy rate; MSR = miscarriage rate; TBSR = total baby sex ratio; LBW = low birth weight rate; BDR = baby with birth defect rate;* *p* < 0.05 **p < 0.01 ***p < 0.001

Using the chi square test for independent samples, the comparison of clinical outcomes between Group IVF/H and Group IVF/D is shown in Figs. 2 and 3. Compared with the D/ET subgroup using frozen sperm (donor sperm) fertilization, the HER was higher than that of the H/ET subgroup using fresh husband sperm (*p* < 0.05). In contrast, the values of the FER, CLR and HER in the D/FET subgroup were lower than those of the H/FET subgroup using fresh husband sperm (all *p* < 0.05). After embryo transfer, the BDR in the D/ET subgroup was lower than that of the H/ET subgroup using fresh husband sperm (*p* < 0.001). Meanwhile, the MUR in the D/FET subgroup was lower than that of the H/FET subgroup using fresh husband sperm (*p* < 0.05).

### Parental factors exposure to frozen sperm fertilization and frozen embryo transfer: the comparison of clinical outcomes between Group IVF/H and Group IVF/D

When both frozen sperm and frozen embryo transfer were used in the D/FET group, the HER was higher than that for the use of both fresh sperm and fresh embryo transfer in the H/ET group (*p* < 0.05). After embryo transfer, the CPR and LBR values in the D/FET subgroup were higher than those in the H/ET group (*p* < 0.001). Meanwhile, the LBW in the D/FET subgroup was higher than that in the H/ET subgroup (*p* < 0.05).

## Discussion

The results of our study indicated that using frozen sperm or frozen embryo transfer have different effects on the different IVF stages, frozen sperm mainly increases fertilization rate and reduces birth defects, while cryopreservation of embryos increases pregnancy rate respectly. For example, in terms of the single factor comparison, the frozen embryo transfer method was more conductive to CPR and LBR, of which increased to 63.2% and 61.1% after using frozen embryo transfer in the H/FET subgroup within IVF/H group. The same results of IVF outcomes were also being found within IVF/D group. In recent years, there has been an accelerating trend toward the use of frozen embryo transfer. Several studies focusing on similar frozen strategies have been conducted in recent years[8, 9, 21, 24, 26]. Recently, Qianqian Z et al. collected a larger general population than other reports to explore the superiority of the freeze-embryo strategy in all IVF/H patients. The live birth rate (LBR) after the first complete IVF cycle was 50.74% in the freeze-embryo strategy. For women who were younger than 31 years old, the LBR of that treatment cycle was 63.81% (95% CI: 62.80–64.80%). The LBR of our results, after using frozen embryo transfer in the H/FET subgroup, was 61.1% (the median age of the treated women was 32 years old). Therefore, our report was extremely close to that of Qianqian Z et al. Although the clinical pregnancy rates and live birth rates after cryopreservation are now meta-analyzed to be close to or even higher than those of fresh cycles[7, 9, 10], singletons born after FET have a higher risk of LBW[19]. In our data, the LBW (including singletons, twins and more) was significantly higher in the H/FET subgroup than in the H/ET subgroup receiving embryo treatment (16.2 vs. 4.9, respectively, p = 0.021).

Other wisely, the frozen sperm fertilization method had an advantage of the HER and BDR, of which increased to 52% and reduced to 0%, only in subgroup D/ET comparing to subgroup H/ET. The differentiated results of IVF outcomes were found in subgroup D/FET comparing to subgroup H/FET. Limited research has been performed on outcomes from IVF treatments with frozen donor sperm [5, 6, 25]. The French Sperm Bank network covers 22 centers of sperm cryopreservation working under the same rules: the CECOS. In this prospective study, 3689 pregnancies achieved after IVF with frozen donor spermatozoa (IVF/D) were followed and reported to the central CECOS between 1987 and 1994. In the prospective CECOS study, the miscarriage rate (MSR) was 21.5%, the multipregnancy rate (MUR) was 29% (including that of twins, triplets and more), the low birth weight rate (LBW) was 4.7%, and the baby with birth defect rate (BDR) was 2.7%. In our study, most of the neonatal characteristics achieved after IVF/D were better, showing a lower MSR (2-2.1%) and MUR (9.2–14.3%) and a higher BDR (0.0–1.0%). The gaps between these two studies are due to the younger donors (the median age was 23 years old) or the rapid development of IVF technology itself in these decades, i.e., using frozen embryo transfer to avoid the deleterious effects of controlled ovarian stimulation on the endometrium [8, 9, 27]. Controversially, the frozen spermatozoa are from donors with normal semen parameters whereas the fresh spermatozoa are from the men in the couples undergoing IVF. Although we improved the inclusion criteria for fresh spermatozoa with normal standard semen parameters. We still cannot excluded impaired sperm genome or proteomic quality by which the couples were diagnosed as infertility [20, 28].

Interestingly, we first found that the exposure to both frozen sperm and embryo treatment had a complementary effect comparing to the use of fresh sperm and embryos. The combined data led to increases in the HER (*p* < 0.05), CPR (*p *< 0.001), LBR (*p* < 0.001) and LBW (*p* < 0.05) after the completion of IVF treatment. The value of HER could derived from the paternal effects of frozen sperm, while the other three values (CPR, LBR and LBW) could derived from the maternal effects of frozen embryo transfer. This complementary effect may be explained by the physiological orientation of sperm and embryos. The sperm initiates the process of fertilization while the embryo plays key role in the pregnancy. Furthermore, the freezing of sperm acts as an artificial selection, eliminating the damage sperm (probably including the defect of acrosomes, sperm membranes or sperm DNA et al.,) [29–32]; and the cryopreservation of embryo is to keep the transplantation the same pace with the growth of the endometrium.

Over the last 70 years, the cryobiology of reproductive cells (sperm and oocytes), embryos, blastomeres, and ovarian and testicular tissue has made rapid progress and has been widely used in human reproductive medicine [22]. Unfortunately, people are only concerned about the survival and viability of cells following the freezing and thawing processes, which could result in a live birth baby. However, little is known about the long-term development of newborns developed from paternal (or maternal) frozen gametes or even the genomic integrity of such frozen cells and tissues [23, 33]. More basic research on the mechanism of the cellular response adaptations to cryopreservation is needed in the future. Eventually, we may uncover some of the cellular defense mechanisms that make cryopreserved sperm/embryos more able to survive.

## Conclusion

This study demonstrates that using frozen sperm or frozen embryo transfer have distinguished effects on the different IVF stages. Especially, the use of frozen embryos and frozen sperm have complementary IVF outcomes. Basic research on the mechanism of the cellular response adaptations to cryopreservation are needed to support our findings.

## Data Availability

The original data of this article cannot be shared publicly for the privacy of couples that participated in the study. Any requests will be considered by the corresponding author. All data generated or analysed during this study are included in this published article.

## Declarations

### Trial registration

N/A.

## List of abbreviations

IVF/H: In vitro fertilization using husbands’ sperm.
IVF/D: In vitro fertilization using donors’ sperm.
FER: Fertilization rate.
CLR: Cleavage rate.
HER: High-quality embryo rate.
BPR: Biochemical pregnancy rate.
CPR: Clinical pregnancy rate.
LBR: Live birth rate.
MUR: Multipregnancy rate.
MSR: Miscarriage rate.
TBSR: Total baby sex ratio.
LBW: Low birth weight rate.
BDR: Baby with birth defect rate.
